# The enigma of inverted lymph node architecture in pigs: insights into immune function and vaccination design

**DOI:** 10.3389/fimmu.2026.1829081

**Published:** 2026-06-10

**Authors:** Thibaut Larcher, Sébastien Picault, Bertrand Bed’Hom, Laurence Dubreil, Xavier de La Bernardie, Isabelle Schwartz-Cornil, Nicolas Bertho

**Affiliations:** 1 APEX, PAnTher, INRAE, Oniris, Nantes, France; 2 BIOEPAR, INRAE, Oniris, Nantes, France; 3 Institut de Systématique, Evolution, Biodiversité (ISYEB), UMR7205, Muséum National d’Histoire Naturelle, CNRS, Sorbonne Université, EPHE, Université des Antilles, Paris, France; 4 UMR 6457, Subatech, Laboratoire de Physique Subatomique et des Technologies Associées, Nantes, France; 5 Université Paris-Saclay, INRAE, UVSQ, VIM, Jouy-en-Josas, France; 6 INSERM, Vaccins Immunopathologie Immunomodulation, Jouy-en-Josas, France

**Keywords:** evolution, lymph node chain, porcine, size, vaccine design

## Abstract

It has been known for decades that pigs, as well as some other mammals, possess a peculiar lymph node architecture commonly referred to as “inverted”. Although some direct consequences of this structure have been described, such as the altered circulation pattern of mature lymphocytes, the overall impact of this inversion on the induction and maturation of the adaptive immune response has never been explored. Here, we tentatively propose a series of hypotheses that could be experimentally tested. If confirmed, these hypotheses might prompt a reconsideration of conventional vaccination strategies in humans and perspectives for possibly improving the outcomes of vaccination in mammals possessing standard lymph nodes.

## 1 Introduction

At the end of the 19^th^ century, Chievitz described the peculiar structure of the porcine lymph nodes (LN), historically referred to as “inverted” (1). Thirty years later, the paucity of lymphocytes in porcine efferent lymph was noticed (2), leading to the observation that, in pigs, immune cells exit the LN through blood vessels (3–5). In mice or humans, immune cells exit the “standard” LN through the efferent lymph (6).

Since that time, three decades later, to our knowledge, we have been the first to implement an experimental research program on the peculiarities of inverted LN using modern tools (7–9). Our goals were to explore the fine structure of inverted LN to infer the potential immunological consequences of this inversion.

a) Structure differences.

**Figure 1:**
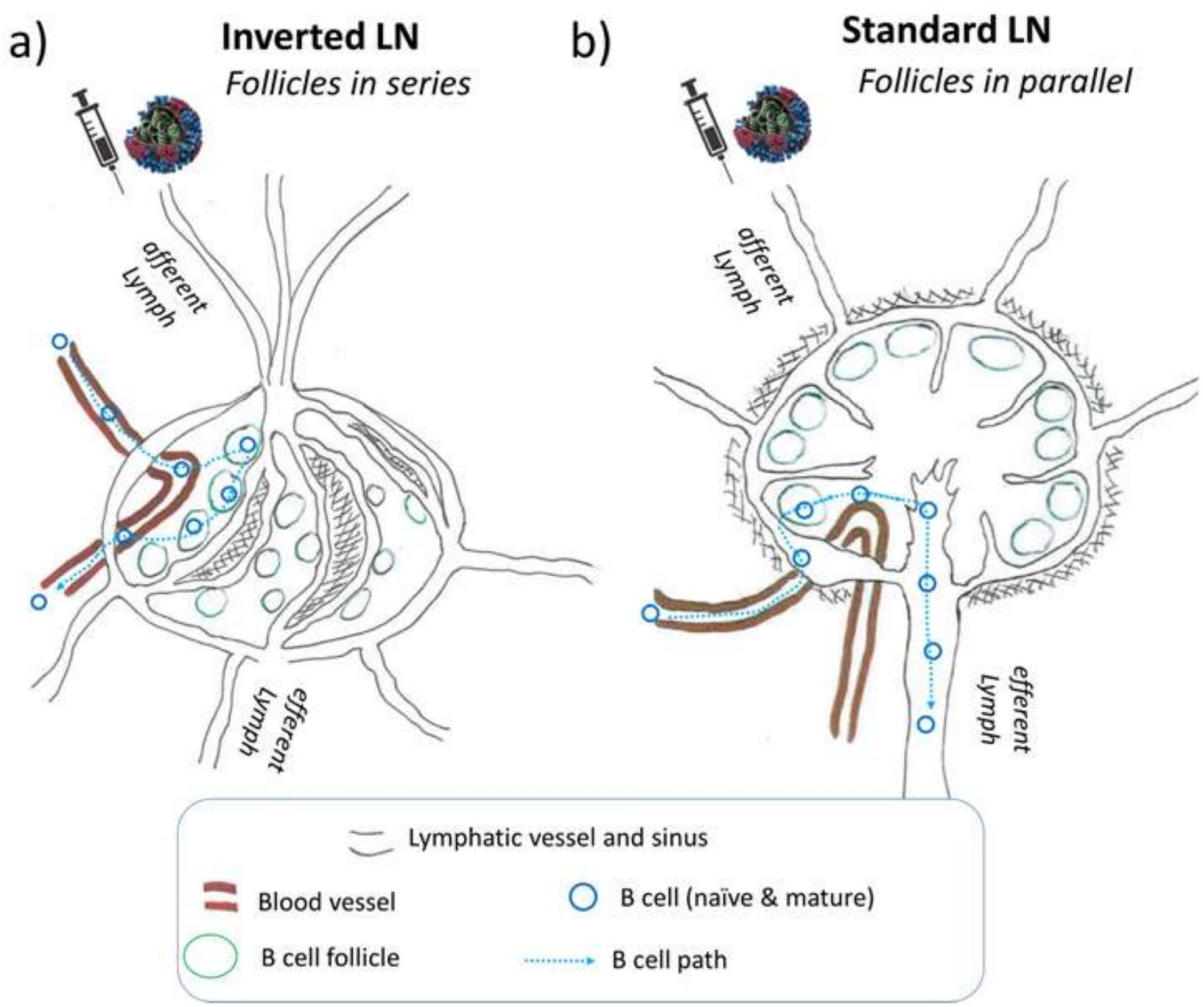
Inverted vs standard lymph nodes: intra-lymph node architecture and B cell circulation. Scheme of **(a)** inverted LN and **(b)** standard LN architectures highlighting the circulation of B cells with their entry in the LN from blood through High Endothelial Venule (HEV) and their exit through either efferent blood vessel (inverted LN) or efferent lymph (standard LN).

When looking at the inverted LN structure and functioning, three points deserved to be highlighted:

(i) In inverted lymph nodes, follicles are predominantly located in the core of the organ (the medulla), aligned along trabeculae composed of dense connective tissue, whereas in conventional lymph nodes, follicles are situated at the periphery (the cortex). Thus, if a LN is considered as a sphere, the space available for follicles in porcine LN may roughly correspond to the volume of the sphere, whereas in human LN, this space is related to the external area of the sphere. Therefore, more room may be available for follicles in inverted LN compared with standard LN.

(ii) In porcine LN, follicles are clearly distributed in several rows, each served by a lymphatic vessel apposed to a trabeculae (5, 8). One afferent lymphatic conduit connects and serves dozens of follicles (9), from the entry to the exit of the LN, in a one-way lymphatic flow. We propose to consider these follicles as connected in series (**Figure 1a**). In standard LN, follicles are distributed along the subcapsular sinus, which is served by different afferent lymphatics, leading to a flow directed toward the medulla, the center of the LN, which is devoid of follicles. We propose to consider these follicles as connected in parallel (**Figure 1b**).

(iii) Finally, in inverted LN, lymphocytes (B and T cells) exit the LN through the blood, whereas in standard LN, lymphocytes exit through the efferent lymphatic vessel, which pours into the next LN of the lymphatic chain. In standard LN species, LN chains can be composed of two to more than ten consecutive LN (10, 11), the efferent lymphatic vessel of the terminal LN dumping into the venous blood system near the heart *via* the thoracic duct, the right duct, or the cervical ducts, depending on the drainage area. Thus, considering lymphatic fluid circulation and lymphatic fluid born antigens transportation, both LN chain types are similar, however considering lymphocyte circulation, standard LN are connected to each other, whereas inverted LN are autonomous.

In conclusion, compared to standard LN, individual inverted LN may have a higher density of follicles that are connected to each other. Conversely, considering lymphocyte circulation, each inverted LN is mostly autonomous from the other LN of the lymphatic chain.

b) Evolutionary repartition.

**Figure 2:**
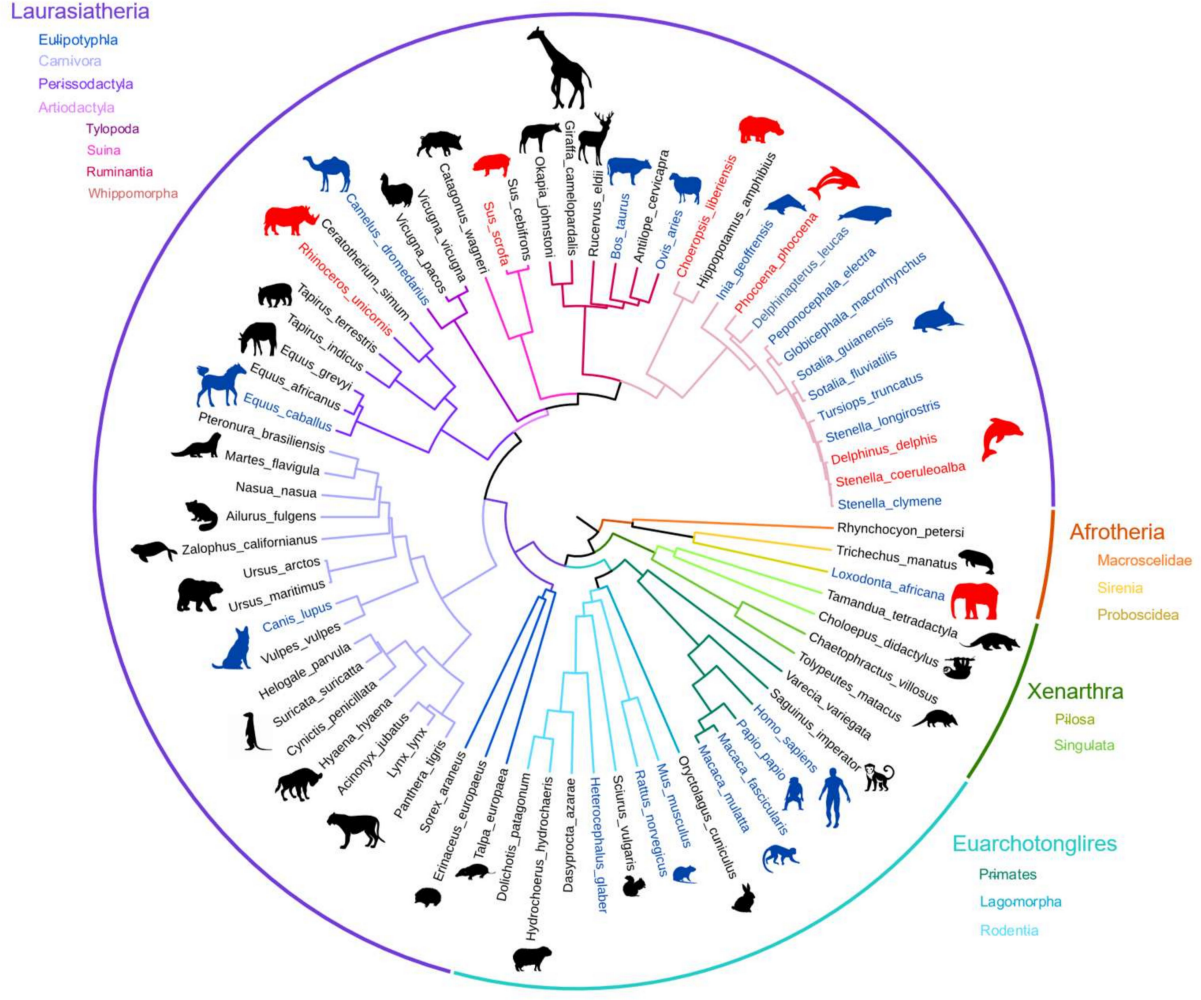
Phylogenetic distribution of inverted and standard lymph nodes in mammals. In blue are depicted species described with standard LN, in red species with inverted LN. In black, some species for which no information are available but might be obtained from French zoological parks collaboration.

As stated by the evolutionary biologist Theodosius Dobzhansky, “*Nothing in biology makes sense except in the light of evolution*” (12). Lymph nodes are found only in mammals and in some birds (from Anseriformes order, also known as waterfowl, such as ducks and swan) (13, 14); however, in birds, they consists of poorly differentiated lymphoid tissue. Accordingly, it is important to explore the distribution of species exhibiting inverted LN across the mammalian evolutionary tree. Two descriptions of marsupial species LN have been published: *Phascogale calura* (Red-Tailed Phascogale) (15) and *Isoodon macrourus* (Northern brown bandicoot) (16), both presented a standard structure. In placental mammals, inverted LN have been so far mainly described (**Figure 2**) in Laurasiatheria and Afrotheria with an uneven occurrence. For Laurasiatherian, in the Whippomorpha suborder, only some Odontoceti (such as *Delphinus delphis*, *Phocoena phocoena*, *Stenella coeruleoalba* (12–17)) present inverted LN. To our knowledge, no data are available for Mysticeti (baleen whales), but some Hippopotamidae (accessible primary publications are not available) have been described with inverted LN. Moreover, some Suina (swine) (1), but neither Ruminantia (18), nor Tylopoda (camels) (19) studied species present inverted LN. In the Perissodactyla lineage, Rhinocerotidae (20, 21), but not Equidae species present inverted LN. To our knowledge, no data are available for Tapiridae. In other mammalian lineages, inverted LN have only been described in Proboscidae (elephants), from Afrotheria superorder (20, 22). To our knowledge, no data are available for other Afrotheria species (including Sirenia, Hyracoidea, Tubulidentata and Afroinsectivora). Finally, no data are available for Xenarthra, the fourth mammalian superorder, which encompasses armadillos (Cingulata), anteaters and sloths (Pilosa).

This quick overview underscores the need to define more precisely the extent and distribution of LN inversion. While initiating a program to collect and analyze LN from various mammalian species to improve our understanding of LN architecture, it also became evident that clear criteria for distinguishing between standard and inverted LN are not yet established. In addition, more informative visualization techniques would be of interest to establish unambiguous criteria for LN structural attribution. In any case, according to the data available to date, it appears that the ancestral state is the standard LN, and inversion occurred independently in Laurasiatheria and Afrotheria lineages, but not in the Euarchontoglires super-orders.

One possible relationship between species presenting inverted LN is their hair scarcity. This might point to the inactivation of a pleiotropic gene that would have an impact on the skin (such as the absence of sebaceous glands probably related to inactivation of MC5R gene (23)) and on the embryonic development of LN. Arguing against this possibility are our own species *Homo sapiens*, as well as personal observations of the naked mole rat (*Heterocephalus glaber)*, both belonging to Euarchontoglires, which present standard LN.

Another possible link between species presenting inverted LN is that, with the exception for pigs, all known species with inverted LN are large animals (more than 3 meters in body length). However, body size in animals is highly variable across evolutionary timescales and clades. There have been several convergent evolutions toward large body size (24, 25): genetic mechanisms selected for a particular function may have pleiotropic consequences, meaning they can also affect other functions; alternatively, the same type of adaptation can have different origins: this is the case for the loss (or reduction in density) of hair in large terrestrial and aquatic mammals (thermoregulation *vs* hydrodynamics). It is also worth noting that some large animal species (some Odontoceti, for instance *Tursiops truncatus*, *Sotalia guianensis*, *Sotalia fluviatilis*, *Stenella clymene*, *Stenella longirostris*, *Inia geoffrensis*, *Peponocephala electra*, *Globicephala macrorhynchus*, *Delphinus delphis*, *Delphinapterus leucas* (17, 26–29)) do not possess inverted LN.

It must be clarified that in species with inverted lymph nodes, despite the absence of lymphocyte recirculation between lymph nodes within the same chain, lymphatic chains are still present, and inter-nodal connections are preserved allowing lymph fluid circulation through lymphatic vessels. This conserved lymph flow implies continuity of antigen transport from one lymph node to the next along the chain (spillover), but it presents also non-immune functions. Indeed, the lymphatic network is involved in fluid drainage, which is essential for the reabsorption of excess interstitial fluid and proteins and their return to the bloodstream, thereby preventing oedema and maintaining tissue pressure and homeostasis (25). We may hypothesize that the conservation in inverted LN species of LN positioning on this network as well as of antigen transportation from one LN to the next may simply account for embryologic development constraints.

To note, the pig presents three other peculiarities (30): i) it is a high γδ T cells species (31) like Bovidae (cow and sheep) (32, 33); ii) it presents pulmonary intravenous macrophages (PIM) similarly to a majority of Laurasiatheria (34), including cetaceans (35); finally, iii) in pig the majority of extrathymic regular memory/activated CD4 T cells expressed CD8α (36). No other species have been reported so far with this extended CD8α expression. To our knowledge none of these features are evolutionary related nor present apparent link with LN inversion.

In conclusion, according to the known distribution of inverted LN in the mammalian evolutionary tree, multiple criteria might be considered: not belonging to Euarchontoglires, having few hairs, and/or being large.

## 2 Hypothesis

Compared with standard LN, individual inverted LN are expected to display a higher density of follicles. Moreover, with respect to lymphocyte circulation, each inverted LN is autonomous from the other LN in the lymphatic chain. Given that inverted LN are mostly present in large species, we propose a hypothesis that LN inversion has been convergently selected in some large species, allowing a faster circulation and redistribution of patrolling naïve and mature lymphocytes.

In standard LN, the lymphocyte journey in the lymphatic system from a LN to the next LN in the chain is a slow process within a low-pressure lymphatic circulatory system. A naïve B cell that does not encounter its cognate antigen may reside for 10 to up to 70 hours in a single LN (37, 38). Thus, patrolling throughout three or more LN of a complete lymphatic chain may take several days, thereby delaying the full amplification of the adaptive immune response during infection. In inverted LN, naïve B cells exit directly into the blood. Once in the bloodstream, lymphocytes require only a few minutes to recirculate through the entire high-pressure circulation (37), and may rapidly re-enter a new LN or other lymphoid organs such as the spleen and mucosal-associated lymphoid tissues for further rounds of amplifications and repertoire shaping.

Similarly, in large species with inverted LN the release of activated lymphocytes directly into the bloodstream may shorten the delay between their activation in the LN and their recruitment to infected, inflamed tissues, enabling them to combat the pathogen. To note, a similar function (39) may be attributed to hemal nodes (40) described in Bovidae and Cervidae. Finally, compared with standard LN, inverted LN may display a higher follicular capacity, which could result in enhanced local B cell activation and maturation within a single inverted LN.

In addition to these straightforward considerations, we would like to further explore the implications of the differential follicular wiring in series or in parallel in inverted and standard LN, respectively. It has been observed in mice that memory B cells generated after primary vaccination remain present in their LN of origin and can re-enter follicles upon antigenic recall, for a new round of mutation and selection, leading to increased affinity (41). Moreover, in human tonsils and LN, a unique memory B cell clone can be present in several follicles (42–45).

**Figure 3:**
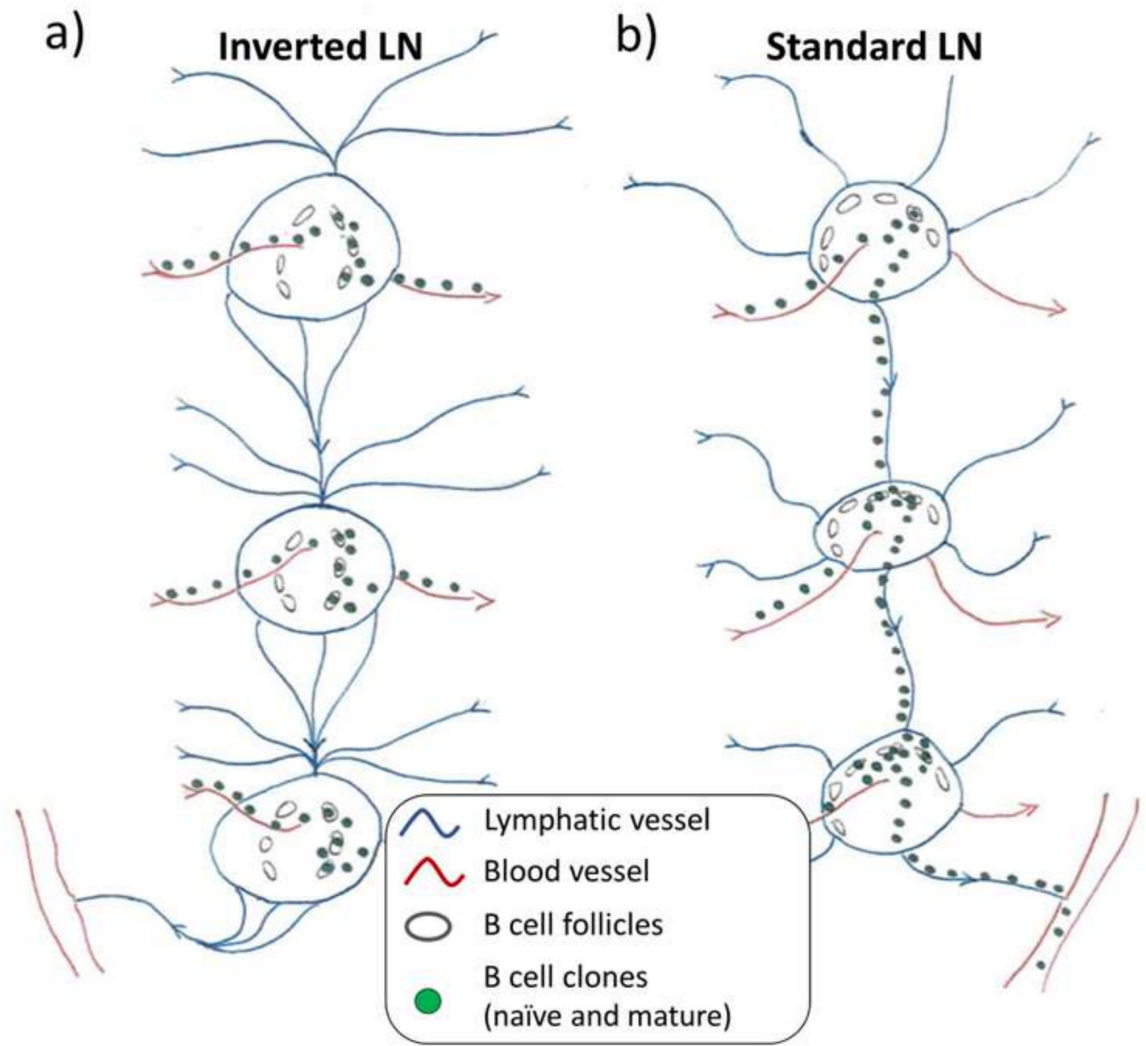
B cell circulation in inverted **(a)** and standard **(b)** lymphatic systems.

According to these data, our additional hypothesis is that, during the same ongoing immune response, early memory B cells originating from the same parental clone, can re-enter follicles and be reactivated by their cognate antigen to undergo a new round of affinity maturation through mutation and selection. In inverted LN, serially connected follicles would allow frequent re-entry into the next follicle along the lymphatic sinus (**Figure 3a**), whereas this would be rare in the parallel connected follicles of standard LN (**Figure 3b**). Conversely, in standard LN chains, this process might occur across successive LN, which is not possible in inverted LN chains, since mature B cells exit the LN directly into the blood stream. However, in standard LN, this process is likely to be slower and less frequent because, in addition to specific B cells, antigens must also flow in sufficient amounts from the first afferent LN to its connected efferent LN. In brief, the possibility of affinity maturation along the LN chain in standard LN may have been replaced in inverted LN by the distribution of GCs in series along the same lymphatic flow, inside a single LN.

To sum up, compared with standard LN, (i) inverted LN present a higher follicle capacity, and (ii) their structure may allow efficient intranodal, multi-follicular maturation of B cells, independently of the LN chain. Thus, species with inverted LN may exhibit greater autonomy of individual LN, which could be advantageous in large species by avoiding the loss of antigens and lymphocytes within the lymphatic system as they travel to the next LN in the chain. This process would allow the rapid release of activated lymphocytes directly into the bloodstream, without having to traverse the entire LN chain, what may take days in large animals. On the other hand, we cannot propose any meaningful hypothesis linking hair scarcity to lymph node inversion, except for a possible indirect association through body size, as large animals often exhibit reduced hair coverage, helping with thermoregulation of terrestrial mammals, or improving hydrodynamics of cetaceans.

## 3 Discussion and perspectives

a) Moving beyond the hypothesis.

We have established a collection network involving French zoological parks, as well as marine mammals collected through the French *Réseau National Échouages* (RNE, PELAGIS). This network provides us with LN from several species, allowing us to broaden our knowledge of species that do or do not present inverted LN. This approach might generate new hypotheses regarding the evolutionary pathways leading to the development of inverted LN.

We are currently developing a mathematical modelling of the B cell maturation/activation process, considering whether LN are standard or inverted, as well as the type of LN chain connectivity. This will allow us to test our hypothesis and to identify experimental settings that will be used to challenge it.

Moreover, one of our strongest statements is that follicles in series may be functionally connected through B-cell migration, making iterative rounds of mutation and selection possible as plasma cells transit through consecutive follicles during the same ongoing immune response. To demonstrate this assertion, we propose, following influenza vaccination of naïve pigs, to separately collect consecutive follicles served by the same sinus using laser microdissection, followed by BCR sequencing. This approach would allow us to demonstrate the presence of B-cell clones derived from an upstream (afferent) follicle to a downstream (efferent) follicle during the same immune response. Of note, we may assume that T-cell maturation follows a similar pattern to B-cell maturation. However, given the more diffuse organization of T-cell areas within LN, especially in inverted LN, it may be more difficult to define “consecutive” T-cell areas and thus to demonstrate a comparable iterative spatial maturation process.

b) From porcine inverted LN to human vaccination.

During our exploration of the peculiarities of inverted LN, we realized that LN chains have been largely overlooked in the literature, with the exception of medical studies on tumor metastasis (46). To our knowledge, over the last decade, only two publications have addressed the immune implications of LN positioning within connected chains. The first study focused on antigen drainage (10). The second demonstrated that T cells activated in upstream LN were more prone to differentiate into effector cells, whereas T cells activated in downstream LN of the same chain were more likely to differentiate into memory cells (47). The authors hypothesized that these functional differences between upstream and downstream LN result from differential biodistribution of antigens and agonists. Interestingly, based on LN architecture, such gradients and spatially driven functional specializations would be expected to be recapitulated within a single inverted LN.

It has recently been shown that the method of antigen delivery can be critical for the development of neutralizing antibodies (45, 48, 49). Interestingly, van Reeth et al. (49) explored the possibility of inducing a pan-H1N1 antibody response in pigs through sequential vaccination with antigenically divergent H1N1 strains, with the aim of promoting secondary immune responses against epitopes conserved between heterologous strains and thereby broadening the anti-HA antibody repertoire. They demonstrated that a broader neutralizing antibody response against three influenza virus strains can be achieved using a sequential vaccination strategy. This protocol consists of vaccination with a first strain at week 0, followed by a second strain at week 4 and a third strain at week 10, rather than three vaccinations with a mixture of the three strains. These data strongly suggest that to achieve a broadly neutralizing status, it is preferable for a B-cell clone to encounter three related antigens successively rather than being exposed to all three antigens simultaneously, and this may hold true in both inverted and standard LN species.

**Figure 4:**
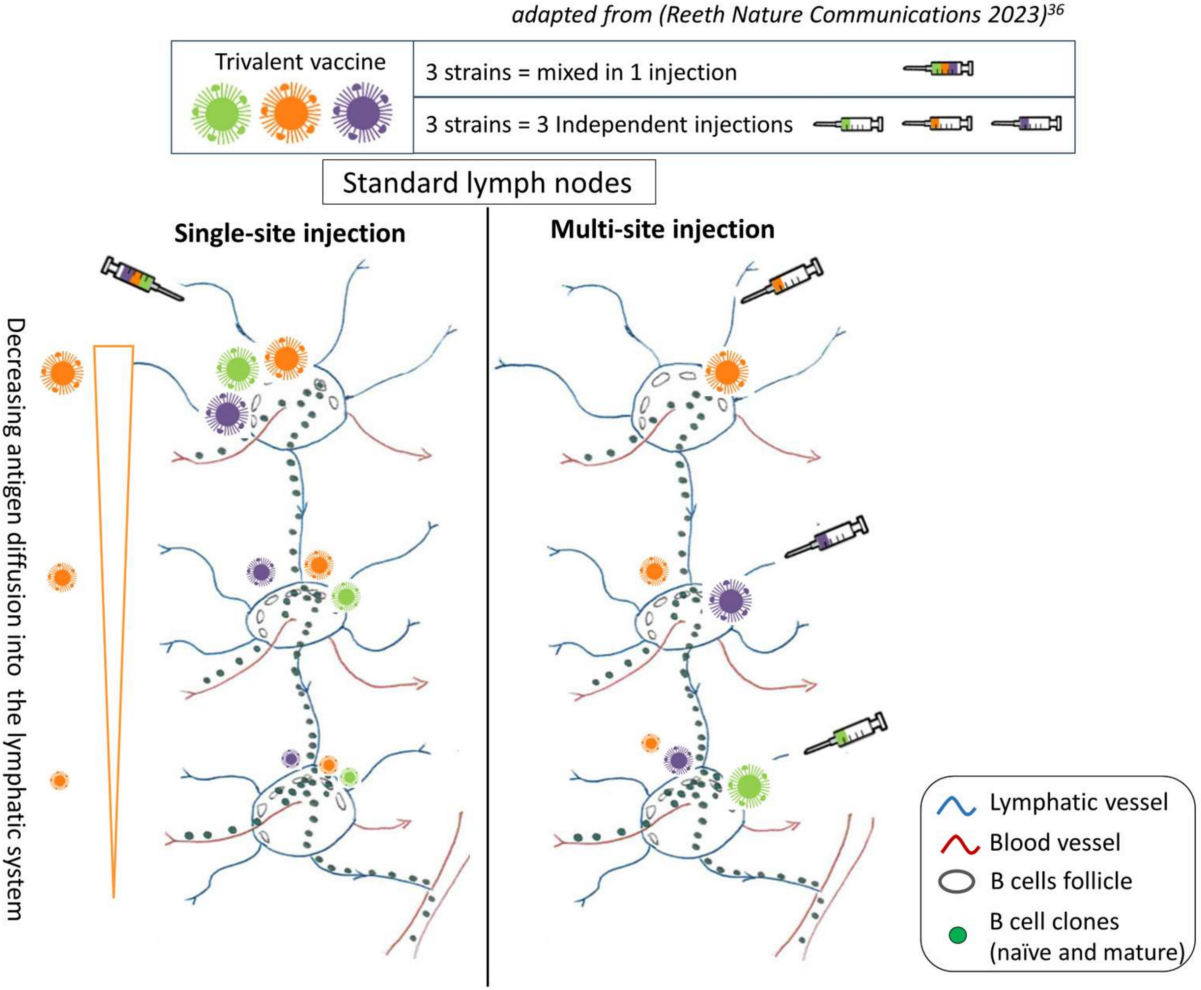
Classical single-site vs spatial multi-sites trivalent vaccination strategies in standard lymph node species.

Taking into account the connectivity of LN chains in standard LN, we propose to transform the chronological, single-site vaccination protocol described by van Reeth et al. (49) into a spatial, multi-site vaccination strategy, which would only apply to species with standard LN. This approach would exploit the capacity of B cells to travel from one LN to the next in standard LN species. It will involve the simultaneous injection of the three influenza vaccine strains at three distinct locations within the same LN chain, thereby targeting three consecutive LN (**Figure 4**).

Based on lymphocyte flow from afferent to efferent LN, and on the possibility for a B-cell clone to visit different follicles successively, this strategy might allow the induction of broadly neutralizing antibodies in a single vaccination session, albeit at the inconvenience of three injections. Different LN chains should be tested in the target species using model antigens. In human, one could propose intramuscular or intradermal vaccination targeting draining LN along the leg (e.g. popliteal LN for the calf, inguinal LN for the thigh and pelvic or lumbar LN for the gluteal region) or alternatively targeting the arm’s LN (e.g. LN of the upper limb, LN of the lower limb and axillary LN for the shoulder region). If effective, this strategy could be applied to highly variable viruses for which broadly neutralizing antibodies are particularly desirable for effective protection, such as influenza virus, severe acute respiratory syndrome coronavirus 2 (SARS-CoV-2) and human immunodeficiency virus (HIV).

## Data Availability

The original contributions presented in the study are included in the article/supplementary material. Further inquiries can be directed to the corresponding author.
